# Benefits and Disadvantages of Electronic Patient-reported Outcome Measures: Systematic Review

**DOI:** 10.2196/15588

**Published:** 2020-04-03

**Authors:** Jill Meirte, Nick Hellemans, Mieke Anthonissen, Lenie Denteneer, Koen Maertens, Peter Moortgat, Ulrike Van Daele

**Affiliations:** 1 Department of Rehabilitation Science and Physiotherapy Faculty of Medicine and Health Sciences University of Antwerp Wilrijk Belgium; 2 Department of Rehabilitation Sciences and Physiotherapy (REVAKI-MOVANT), Faculty of Medicine and Health Sciences, University of Antwerp Antwerp Belgium

**Keywords:** electronic patient-reported outcome measures, paper-based patient-reported outcome measures, systematic review, advantages, pitfalls

## Abstract

**Background:**

Patient-reported outcome measures (PROMs) are important in clinical practice and research. The growth of electronic health technologies provides unprecedented opportunities to systematically collect information via PROMs.

**Objective:**

The aim of this study was to provide an objective and comprehensive overview of the benefits, barriers, and disadvantages of the digital collection of qualitative electronic patient-reported outcome measures (ePROMs).

**Methods:**

We performed a systematic review of articles retrieved from PubMED and Web of Science. The Preferred Reporting Items for Systematic Reviews and Meta-Analyses (PRISMA) guidelines were followed during all stages. The search strategy yielded a total of 2333 records, from which 32 met the predefined inclusion and exclusion criteria. The relevant ePROM-related information was extracted from each study.

**Results:**

Results were clustered as benefits and disadvantages. Reported benefits of ePROMs were greater patient preference and acceptability, lower costs, similar or faster completion time, higher data quality and response rates, and facilitated symptom management and patient-clinician communication. Tablets were the most used ePROM modality (14/32, 44%), and, as a platform, Web-based systems were used the most (26/32, 81%). Potential disadvantages of ePROMs include privacy protection, a possible large initial financial investment, and exclusion of certain populations or the “digital divide.”

**Conclusions:**

In conclusion, ePROMs offer many advantages over paper-based collection of patient-reported outcomes. Overall, ePROMs are preferred over paper-based methods, improve data quality, result in similar or faster completion time, decrease costs, and facilitate clinical decision making and symptom management. Disadvantages regarding ePROMs have been outlined, and suggestions are provided to overcome the barriers. We provide a path forward for researchers and clinicians interested in implementing ePROMs.

**Trial Registration:**

PROSPERO CRD42018094795; https://www.crd.york.ac.uk/prospero/display_record.php?RecordID=94795

## Introduction

In patient-centered care, patient-reported outcome measures (PROMs) are the gold standard for efficiently evaluating patients’ feelings, thoughts, and complaints about a clinical intervention or disease [1].

Clinicians use PROMs to guide and audit routine care and support patient-centered care. Standard intake procedures already include many questionnaires such as generic quality of life questionnaires administered before arthroplastic surgeries [2]. At the patient level, the data can be used to monitor individual progress, investigate the effects of medical and surgical interventions [2], and improve communication between patients and caregivers [3]. On a larger scale, PROM data can be used to screen for health problems, compare outcomes between populations, and assess quality of care. They are widely implemented in clinical research [1,4], with positive effects on patient-clinician communication and mutual decision making. PROMs are traditionally measured using pen-and-paper questionnaires. We aimed to investigate whether pen-and-paper methods are the best option because unsupervised paper-based PROM data collection in clinical trials has resulted in unreadable, missing, or faulty data [5].

The growth of electronic health (eHealth) technologies provide unprecedented opportunities to systematically collect information via PROMs. Patients of all ages and sociodemographic backgrounds worldwide are comfortable using digital networks and services [6]. Furthermore, smartphones and lightweight computers or tablets with touchscreens are omnipresent. Supposed advantages of electronic PROMs (ePROMs) include more complete data capture and lower cost but it is unknown if the advantages of ePROM outweigh the disadvantages. Various research groups in different medical fields have investigated the use of electronic questionnaires in different patient groups; however, the benefits and disadvantages of ePROM collection have not yet been systematically explored. When transferring questionnaires from paper to electronic format, comparability is questioned. Many individual studies and several meta-analyses [7-10] have concluded that scores derived from ePROMs are equivalent to their original paper versions. In other words, scores derived from a computerized measure do not differ from scores derived from the pencil-and-paper version. The International Society for Pharmacoeconomics and Outcomes Research (ISPOR) reported 3 levels of modification (minor, moderate, and substantial) for the migration from original paper-based PROM to ePROM. The ISPOR also provides an effective strategy for testing measurement equivalence (reliability and validity). Minor modification means simply placing a paper-based scale form into a screen-based format without changing font size or altering items. Then, only a cognitive interview with 5-10 patients and a usability test is recommended. Moderate modifications are changes such as splitting single items into multiple screens, requiring the patient to use a scroll bar to see all the items or responses, or changing the order of items. With moderate modifications, equivalence testing with a randomized parallel group or randomized crossover design is advised in addition to usability testing. Major changes include removing items. With major modifications, full psychometric evaluation and large-scale usability testing in the target population are required [11]. However, recent evidence suggests that previous usability evidence in a representative group is sufficient to assume equivalence [12].

The ISPOR’s electronic patient-reported outcome (ePRO) System Validation Task Force also developed recommendations on the validation of electronic systems used to collect PRO data in clinical trials [13]. This report enhances the understanding of different steps needed to develop ePROM. Both reports, based on expert opinion, give important insights in the development of ePROM based on the paper-version counterpart.

Hence, there is growing emphasis on ePROMs with a clear shift towards electronic data capture driven by regulatory and practical considerations [14], and patients seem motivated to use these tools as long as they provide added value and quality of care [15]. While a number of reviews have summarized the equivalence of digital questionnaires, none of these reviews systematically assessed the benefits and disadvantages of ePROM. Since more people have gained access to the internet via many types of devices, many opportunities have arisen in the eHealth ecosystem. Weighing the advantages against the disadvantages is necessary and imperative for clinical practice and research purposes. This systematic review aimed to evaluate the scientific evidence for the use of digital questionnaires to assess PROMs and more particularly describe the benefits and disadvantages.

## Methods

The protocol for this review was accepted in the PROSPERO systematic review database (ID: CRD42018094795) [16]. This systematic review was conducted and reported following the Preferred Reporting Items for Systematic Reviews and Meta-Analyses (PRISMA) guidelines [17].

### Inclusion and Exclusion Criteria

The PICO model was used to define the criteria to assess study eligibility. To be included in this review, studies had to report about questionnaires that evaluated PROMs. These questionnaires had to be in digital format (ie, tablet, computer, or mobile app). The criteria did not include a comparison; both studies comparing digital against paper formats and studies solely reporting about a digital questionnaire were included. The outcome measures described either benefits or disadvantages of digital questionnaires. This systematic review focused on the use of digital questionnaires. The scope of digital questionnaires was broad, including any web-, tablet-, computer-, or mobile-based method to assess PROMs.

To be included, articles had to evaluate ePROMs, preferably those used by general practitioners, doctors, occupational therapists, physiotherapists, or other health care workers; assess questionnaires in a digital format; compare a digital questionnaire with a paper-based method; describe either benefits or disadvantages of a digital questionnaire; or describe a randomized controlled trial or cohort, case-control, longitudinal, descriptive, or qualitative research.

Articles were excluded when the questionnaire was not used in the health care setting, it did not describe one of the listed aspects or clinical parameters mentioned in the keywords, or it described a review, meta-analysis, case study, or case report.

### Information Sources and Search Strategy

A systematic computerized search strategy was performed in PubMed and Web of Science in October 2017. Additionally, manual screening of reference lists of relevant published literature occurred in November 2017. Neither filters nor limitations on the query were used. We searched for articles using the keywords patient related outcomes, self-management, self-reported, self-administered, questionnaire, survey, PRO, ePRO, PROM, ePROM, electronic, web-based, tablet-based, and digital questionnaires in combination with the keywords advantages, disadvantages, benefits, efficacy, acceptability, feasibility, validity, reliability, reproducibility, and response rate.

### Study Selection

Two reviewers (JM and NH) searched and screened the identified records based on the eligibility criteria. Screening and selection were performed first on the title and abstract and second on the full text. Only published full-text articles in English were included.

### Data Collection

The following relevant information was extracted: study description, examined ePROMs, outcome measures, and main results.

### Methodological Quality

Two researchers (NH and JM) independently assessed the methodological quality. Both researchers were not aware of the other’s evaluation before holding a consensus meeting. Methodological quality of the experimental studies was assessed with a 10-item checklist provided by the Dutch Cochrane Centre [18]. Observational studies were assessed with the 14-item Quality Assessment Tool for Observational Cohort and Cross-Sectional Studies [19]. Studies with high methodological quality were given more value when making final conclusions about the advantages and disadvantages of ePROMs.

## Results

### Study Selection

**Figure 1:**
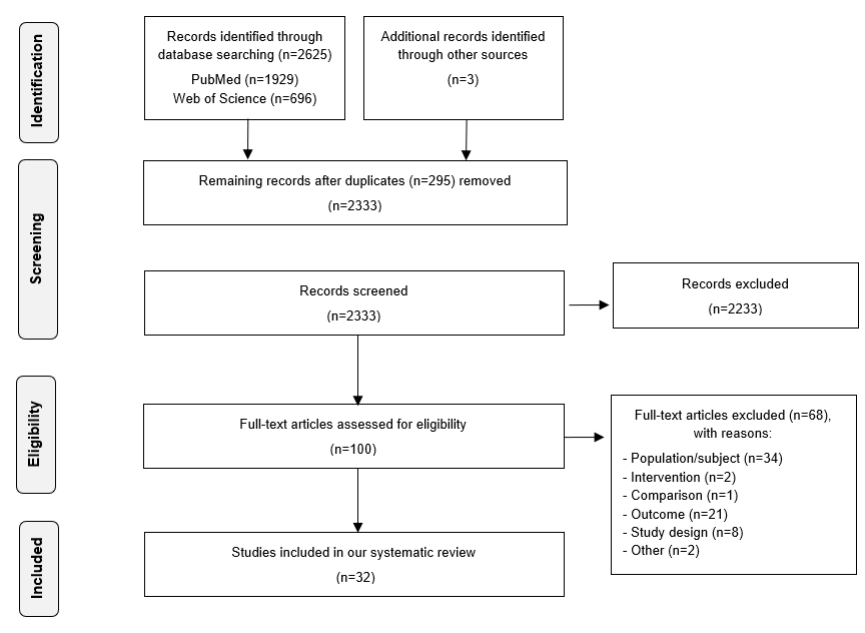
Flow diagram of the study selection process.

The results of the literature search and study selection are shown in Figure 1. In summary, 2333 records were identified after removing duplicates. After screening the titles and abstracts, 100 eligible studies remained, and the full-text versions were screened. After reading the full text, 32 articles that met the predefined inclusion and exclusion criteria were included in this systematic review. Two reviewers (NH and JM) screened the identified records using the eligibility criteria. Screening was first performed based on the titles and abstracts. Full-text articles were retrieved when a record was assessed as eligible. Each full-text article was once again assessed against the inclusion criteria. Disagreements were discussed between the researches, and consensus was always achieved. The intervention of a third reviewer (UVD) was not necessary.

### Study Characteristics

The results of this systematic review are based on 14 observational studies [20-33] and 18 experimental studies [34-51]. The retrieved experimental studies either compared an ePROM

versus a paper-based PROM in two separate groups [23,35,39,41,44,48,50] or compared the two modes of administration within the same groups, after randomizing in which order the modes of administration were completed [36-38,40,42,43,45-47,49,51].

The populations varied from healthy people [31,36,39,44,49] to patients with a certain condition or disease [20-22,24-30,32-35,38,40,41,43,45,51]. We did not differentiate the results by population since the goal was to systematically evaluate all possible advantages and disadvantages of ePROMs regardless of the population. Most articles were found in the field of cancer research (9/32) and musculoskeletal research (10/32).

Overall, the included studies represented 11,006 individuals (mean age 49 years, range 13-93 years) exposed to an ePROM or asked their opinion about it. Not all studies [30,31,38,51] reported the ratio between male and female participants, meaning the sex of 3038 of the 11,006 participants was unknown. Based on the available data, 61% (4827/7968) of the subjects were female, and 39% (3141/7968) were male.

The different ePROM modalities were personal digital assistants (2/32, 6%), smartphones (2/32, 6%), tablets (14/32, 44%), computers (9/32, 28%), or not specified (5/32, 16%). Web-based systems were used the most (26/32, 81%).

The characteristics of the studies are presented in Table 1, and the results are presented in Table 2.

**Table 1:** Characteristics of the studies included in the systematic review.

Study	Risk of bias score^a^	Level of evidence^a^	Population	Sample size, n (male/female)	Age (years), mean (range)	Setting
[46]^b^	6/10	A2	Patients with a skin condition	104 (45/59) 57 (29/28; group 1) 47 (16/13; group 2)	51.5 (20-89) 51.5 (19.3^c^; group 1) 51.4 (18.2^c^; group 2)	Outpatient clinic
[20]^d^	9/14	C	Patients post-major gynecologic cancer surgery	49 (0/49)	56 (23-74)	–^h^
[45]^b^	6/10	B	Cardiology patients	–	–	Outpatient clinic
[31]^d^	6/14	C	People from Andalucia	2493	–	At home
[41]^b^	3/10 B	B	Patients who had undergone hand surgery	468 (216/270)	48.3 (18-91)	Private practice
[23]^b^	4/10	B	Patients in a cardiac, pulmonary, occupational, or cancer rehabilitation program	126 (56/70)	56.3	Prior to rehabilitation at home
[49]^b^	7/10	B	Healthy aging adults	49 (13/36)	64 (57-71)	Research center
[28]^d^	10/14	B	Patients with a cancer diagnosis	1484 (607/877)	56.3	Inpatient reference center
[47]^b^	6/10	B	Patients with rheumatoid arthritis	40 (17/23)	65 (44-83)	In the clinic
[26]^d^	9/14	B	Patients with adjuvant and metastatic breast cancer	202 (0, 202)	54 (20-85)	Outpatient visit
[34]^d^	7/14	C	Patients with cancer pain	–	–	Outpatient oncology clinic
[43]^b^	4/10	B	Patients with lung cancer	148 (84/64)	67 (35-81)	Community centers
[29]^d^	9/14	B	Patients with sickle cell disease	15 (9/6)	26 (16-54)	At home
[25]^d^	8/14	C	Patients with multiple sclerosis	55	46.3	At home
[51]^b^	6/10	B	Patients with asthma or rhinitis	116	17-65^e^	Clinic visit
[48]^b^	5/10	B	Patients with THR^f^ or TKP^g^	100 (41/59)	67 (36.7-88)	Outpatient clinic
[32]^d^	7/14	A2	Patients with THR or TKP	565 (198/367; THR) 387 (126/261; TKR)	65.9 (10.6^c^; THR) 68.9 (9.7^c^; TKR)	At home
[50]^b^	8/10	A2	Healthy women referred for mammography	533 (0/533)	20-67^e^	At home
[21]^d^	7/14	C	Patients with epilepsy	502 (272/230)	27.98 (15-73)	At home
[44]^b^	7/10	A2	Healthy adolescents	591 (272/319)	14 (13-17)	At school
[27]^d^	9/14	C	Geriatric patients (>70 years) with gastrointestinal cancer	37 (17/20)	77 (70-89)	Outpatient institute
[39]^b^	7/10	A2	Adolescents	933 (432/501)	14.7 (13-17)	At school
[33]^d^	9/14	C	Ambulatory neurological patients	323 (134/190)	32.2	Ambulatory clinic
[42]^b^	6/10	A2	Patients with rheumatoid arthritis, lupus, or spondyloarthritis	153 (47/106)	45.7	Outpatient care center
[40]^b^	6/10	A2	Patients with rheumatoid arthritis	87 (29/58)	14.7 (34-83)	Outpatient clinic
[37]^b^	6/10	B	Patients with axial spondyloarthritis	55 (45/10)	51 (34-63)	Outpatient clinic
[30]^d^	6/14	C	Dialysis patients	66	66 (36-91)	Home dialysis units
[22]^d^	10/14	C	Patients with HIV	42 (28/14)	50 (26-66)	Outpatient clinic and at home
[35]^b^	7/10	A2	Orthopedic patients (upper extremity, spine, or arthroplasty)	483 (235/248)	55.7 (14-93)	Three subspecialty services during outpatient visits
[38]^b^	6/10	A2	Patients from an orthopedic clinic (spine, upper extremity, and trauma)	308	–	Outpatient clinic
[36]^b^	7/10	A2	Healthy volunteers	147 (68, 79)	62.7 (49-75)	At home
[24]^d^	8/14	C	Cancer patients	158 (116/42)	51.9 (22-81)	clinic and home

^a^Based on the Dutch Centraal BegeleidingsOrgaan-classificatiesysteem (CBO) [52].

^b^Experimental study.

^c^Mean (SD).

^d^Observational study.

^e^Range.

^f^THR: total hip replacement.

^g^TKR: total knee replacement.

^h^Not applicable.

**Table 2:** Results of the studies included in the systematic review.

Study	Electronic delivery method		ePROM^a^, outcome, and results		
	Web/PC^b^	Device			
[46]	Web	Tablet	**DLQI^c^**		
				Preference	76% prefer electronic
				Completion time	Electronic took 9 s longer than pencil and paper (*P*=.008), older participants took longer (*r*^2^=.257, *P*=.012)
				Agreement	ICC^d^=.98, CI 0.97-0.99
[20]	Web	–	**EORTC^e^, QLQ-C30^f^**		
				Completion rate	92% completed the first measurement, 74% completed the 6-month measurement, 82% completed ≥4 of 7 sessions
				Satisfaction and other outcomes	92% found it easy to use, 85% continued using it, 85% recommended it
[45]	Web	PC	**SAQ^g^, SF-36^h^**		
				Preference	82% preferred electronic, there was no effect on preference with age, sex, race, computer use, education, visual impairment, or reading level
				Completion rate	No differences in the completion rate
				Completion time	SAQ completion time: 5.53 min electronic, 4.78 min paper (*P*<.05); SF-36 completion time: 6.76 min electronic, 5.44 min paper (*P*<.05); the log-on procedure was not significantly different
				Agreement between electronic and paper	For the 5 SAQ domains *r*=0.84-0.93; for the 8 SF-36 subscales: *r*=0.54-0.75
[31]	Web	PC	–		
				Preference	83.6% preferred pencil and paper, 14.4% preferred internet
				Data completion	Unanswered questions: 9.3% pencil and paper, 4.9% internet (==*t* =14.85, *P*=.01)
				Data missing	Internet answers were more detailed than pencil and paper answers in 4 of 5 questions (*P*<.05)
[41]	Web	Tablet	**DASH^i^**		
				Data completion	24% of questions were unscorable with pencil and paper, compared with 2% for electronic (*P*<.001); electronic was more likely to be scorable (OR^j^=13.5, *P*<.001)
				Data missing	Mean (SD) of 2.6 (4.4) with pencil and paper vs 0.1 (0.8) with electronic (*P*<.001), electronic format had an inverse relationship with omitted questions (beta=–0.358, *P*<.001)
[23]	Web	–	**PAM-13^k^, MacNew^l^, FQ^m^, EORTC, QLQ-C30, HADS^n^**		
				Demographic factors	Preferred electronic over paper: younger age (*P*=.008), married/cohabitating (*P*=.004), internet available (*P*<.001), educated (*P*=.092)
				Preference	77.8% prefer web-based forms
				Completion time	Web-based, ~9.5 min; paper-based, ~24 min
				Data completion	Inadequate responses did not exist for the web version due to the system design
				Data missing	Fewer total data points missing on paper-based forms than on web-based forms (*P*<.001)
[49]	Web	Tablet	**PASE^o^, BARSE^p^, PSQI^q^**		
				Demographic factors	Factors affecting preference of electronic vs paper: daily computer use, perceived ease of use, reported anxiety while completing the digital questionnaire (all *P*<.05)
				Preference	Electronic preferred over pencil and paper (z=4.96, SE 3.428, *P*<.001)
[28]	PC	Tablet	**EORTC, QLQ-30**		
				Completion rate	Completion rate 43%-58% from 2005-2010, <20% since 2011 (ePRO^r^)
				Adherence and compliance	Pencil and paper associated with non-completion (OR=2.72, *P*<.001) and poor adherence (OR=2.23, *P*<.001), male sex associated with poor adherence (OR=1.69, *P*=.010)
[47]	PC	PC	**RAQoL^s^**		
				Satisfaction	Electronic > P-P (*P*=.003)
				Preference	64% prefer electronic
				Completion time	Pencil and paper, 6 min; electronic, 5 min *P*=.194
				Agreement between electronic and paper	ICC=.982
[26]	Web	Tablet	**EORTC, QLQ-C30**		
				Attitude/ willingness	92.3% of those exposed to both electronic and paper vs 59% of those exposed only to paper (*P*=.001) were willing; patients exposed only to paper more likely to report barriers: data privacy (*P*=.003), technical knowledge (*P*=.02), discomfort using technology (*P*=.02), no internet (*P*=.05)
[34]	Web	Tablet	–		
				Adherence	Patient adherence: 76.8% for pain monitoring, 50.4% for medication monitoring, and 100% for education
				Satisfaction	Limited effort, comfortable, education session appreciated, added value with self-management, medication overview with reminders was supportive
				Experience	Measured using a Likert scale, mean (SD): learnability, 4.8 (0.4); usability, 4.8 (0.5); desirability, 4.6 (0.4); and would recommend app, 4.8 (0.4)
[43]	PC	PDA^t^	**LCSS^u^**		
				Satisfaction	98% of patients reported it acceptable and easy to use, 80% learned it in <3 minutes, 100% of nurses and 86% of physicians said it’s easy to use
				Completion time	Electronic, 2.2 min; pencil and paper, 3-5 min
				Agreement between electronic and paper	Pearson *r=*0.92, ICC=.92, Lin's CCC^v^=.92
[29]	Web	iPhone, iPad, or iPod	**Pain VAS^w^**		
				Completion rate, adherence, compliance	Compliance decreases over time, >35 years old had increased compliance (*P*<.05), compliance greater with iPad than iPhone (*P*<.0025), technical difficulties decreased compliance (*P*<.0025),
				Demographic factors	Information technology comfort level had no impact on adherence
				Agreement	iPhone, ICC=.99 (95% CI 0.92-1.00); iPad, ICC=.97 (95% CI 0.88-0.99)
[25]	Web	–	**MSIP^x^, MSQoL-54^y^, MFIS-5^z^, LMSQoL^aa^**		
				Other symptom insights	46% have greater insights into symptoms; 18% feel better able to handle symptoms; 65.4% feel it’s important for other health care professionals to have access; advantages include availability, overview of symptoms, gain insights, forced to reflect, look back on history; disadvantages include it’s tiring, lot of work, complicated, repeated questions, grammatical errors, no space for free text, monthly completion, login problems, not used friendly, data aren't used by physician
[51]	Web	PDA	**AQLQ^bb^, ACQ^cc^, RQLQ^dd^**		
				Agreement between electronic and paper	AQLQ (*P*=.009), ACQ (*P*=.12), RQLQ (*P*=.05)
[48]	Web	Tablet	**WOMAC^ee^, FJS-12^ff^**		
				Completion time	WOMAC: pencil and paper 170 s, electronic 117 s (*P*<.001); FJS-23: pencil and paper 22 s, electronic 37 s (*P*<.001)
[32]	Web	–	**SF-36**		
				Preference	THR^gg^ 81.8% preferred pencil and paper (CI 78.8-84.7), TKR^hh^ 86.8% preferred pencil and paper (CI 83.1-89.8)
				Demographic factors	Preferred electronic over paper: younger age (*P*<.001), male sex (*P*<.001), higher education level (*P*<.001), higher BMI (*P*=.004)
[50]	Web	PC	**SF-36, MFI-20^ii^, HADS**		
				Completion rate	73.2% with pencil and paper vs 17.9% with internet: difference of 55.3 (48.3-62.3); after a reminder: 76.5% with pencil and paper vs 64.2% with internet: difference 12.2 (4.5-20)
				Preference	55.4% prefer pencil and paper
				Data completion, missing data	63.4% data completion with pencil and paper vs 97.8% with internet (*P*<.001): difference 34.5 (26.6-42.3)
[21]	Web	Smartphone	**MMAS-8^jj^**		
				Demographic factors	Preferred electronic over paper: younger age (*P*=.002), live in the city (*P*<.001), higher education level/stable employment (*P*<.001), more seizures (*P*=.01), lower medication adherence and own a smartphone (*P*=.001)
				Attitude/willingness	65.5% would use it if it was free, 72.3% if it was easy to operate, 59% think it decreases medical visits and related costs, 71.7% say privacy must be protected
[44]	Web	PC	**KIVPA^kk^**		
				Preference	Mean (SD) pleasantness: 2.7 (0.9) for pencil and paper vs 3.0 (0.8) for internet (*P*<.01); mean (SD) difficulty: 3.6 (0.7) for pencil and paper vs 3.9 (0.7) for internet (*P*<.01)
[27]	PC	Tablet	**CSGA^ll^**		
				Feasibility in older patients	≥50% unable complete without assistance (reason: computer illiteracy)
[39]	Web	PC	**CHQ-CF^mm^**		
				Data completion, missing data	0.54% with paper vs 0.04% with internet (*P*<.01)
[33]	Web	PC and tablet	**EQ-5D^nn^, PHQ-9^oo^**		
				Satisfaction	92.3% found it easy to use, 87.6% thought it time appropriate, 77.3% saw a perceived benefit
				Other factors affecting perception of benefit	Provider review (OR 6.56, *P*<.001)
[42]	Web	Tablet	**FFbH^p^, BASDAI^qq^, SF-36**		
				Experience	Older age requires more support
				Preference	62.1% prefer electronic, especially those of younger age and with increased computer knowledge (*P*<.01)
				Data completion	Significantly greater with electronic
				Agreement between electronic and paper	*r*=0.87-0.98; *P*>.05
[40]	PC	PC	**VAS GH, VAS Pain, VAS PGA^rr^, ROAD^ss^, TJC^tt^**		
				Preference	86% prefer electronic
				Completion time	Electronic 7.3 min, pencil and paper 7.9 min (*P*=.006); older age requires greater time for both (electronic: *P*=.02, pencil and paper: *P*=.005)
				Agreement between electronic and paper	No difference between methods and high correlation (all *P*>.05, CCC>.849)
[37]	PC	Tablet	**BASDAI, BASFI^uu^, NRS^vv^**		
				Preference	83.4% prefer the tablet
				Completion time	Tablet 5.1 min, paper 7.9 min (*P*=.04)
				Agreement	ICC>0.9 (*P*<.0001)
[30]	Web	Tablet	**KDQOL-36^ww^, ESAS^xx^**		
				Logistics	Internet/cellular access, link to electronic health records
				Infection control	Hand sanitizer, stylus
				Financials	Financial support necessary?
				Design	Minimalistic, large font, black writing on white background, no distracting graphics, adapted to population
[22]	Web	–	**Symptom self-management tool for PLWH^yy^**		
				Symptoms diminish with targeted strategies	Decreased frequency (effect size=.37) and intensity (effect size=–8.41) over time for all symptoms except diarrhea
[35]	Web	Tablet	**EQ-5D, ODI^zz^, NDI^1^, HOOS^2^, KOOS^3^, QuickDASH^4^**		
				Completion rate	No differences in unanswered questions (*P*>.05)
				Preference	Satisfaction similar; however, 41.4% prefer the tablet (*P*<.001); total 60.38%
				Data completion	No difference in completion rate (*P*=.208)
				Completion time	No difference in the completion time (*P*>.05)
[38]	Web	Tablet	**PSS^5^, FFI^6^, ODI**		
				Preference	68% prefer electronic
				Data completion	Pencil and paper 14 times greater completion (PSS, *P*=.008), 260 times greater completion (FFI, *P*<.001), 11 times greater completion (ODI, *P*<.001)
				Agreement between electronic and paper	Differences in patient-reported outcomes scores not significant (*P*>.05)
[36]	Web	PC	**Nutrinet Sante**		
				Preference	92.2% prefer web; web considered more acceptable (*P*=.002) and with fewer barriers (*P*=.03)
				Data completion	No data missing in web
				Completion time	No significant differences in completion time
				Cost	For a cohort of 500,000 subjects: paper €4,965,833 (€9.94/subject); web-based tool €150,000 (€0.3/subject)
				Agreement between electronic and paper	Agreement ICC=.86-1.00 qualitative variables; ICC=.69-1.00 for 18 qualitative variables (height, weight, hip circumference, waist circumference were all different)
[24]	Web	Tablet	**EORTC**		
				Preference	65.98% prefer electronic
				Habits and attitudes	64.4% of the clinic ePROM group and 91.1% of the home ePROM group found it useful and adequate for QOL; 82.2% would appreciate discussing results with a physician
				Feasibility and suggestions	Perceived benefits included that it was always available, feeling well cared at home, and low cost; the disadvantages included that it was too impersonal and technical issues; suggestions included adjustable font size

^a^ePROM: electronic patient-reported outcome measure.

^b^PC: personal computer.

^c^Dermatology Life Quality Index.

^d^ICC: interclass correlation coefficient.

^e^EORTC: EORTC: European Organization for the Research and Treatment of Cancer.

^f^QLQ-C30: Quality of Life Questionnaire Core 30.

^g^SAQ: Seattle Angina Questionnaire.

^h^SF-36: Short Form-36.

^i^DASH: Disabilities of the Arm, Shoulder, and Hand.

^j^OR: odds ratio.

^k^PAM-13: Patient Activation Measure short form.

^l^MacNew: MacNew Heart Disease Health-related Quality of Life questionnaire.

^m^FQ: Fatigue Questionnaire.

^n^HADS: Hospital Anxiety and Depression Scale.

^o^PASE: Physical Activity Scale for the Elderly.

^p^BARSE: Barriers Self-Efficacy Scale.

^q^PSQI: Pittsburgh Sleep Quality Index.

^r^ePRO: electronic patient-reported outcome.

^s^RAQol: Rheumatoid Arthritis Quality of Life Questionnaire.

^t^PDA: personal digital assistant.

^u^LCSS: Lung Cancer Symptom Scale.

^v^CCC: concordance correlation coefficient.

^w^VAS: visual analogue scale.

^x^MSIP: Multiple Sclerosis Impact Profile.

^y^MSQoL-54: Multiple Sclerosis Quality of Life-54.

^z^MFIS-5: Modified Fatigue Impact Scale-5.

^aa^LMSQoL: Leeds Multiple Sclerosis Quality of Life.

^bb^AQLQ: Asthma Quality of Life Questionnaire.

^cc^ACQ: Asthma Control Questionnaire.

^dd^RQLQ: Rhinoconjunctivitis Quality of Life Questionnaire.

^ee^WOMAC: Western Ontario and McMaster Universities.

^ff^FJS: Forgotten Joint Score.

^gg^THR: total hip replacement.

^hh^TKR: total knee replacement.

^ii^MFI-20: Multidimensional Fatigue Inventory.

^jj^MMAS-8: Morisky Medication Adherence Scale.

^kk^KIVPA: Korte Indicatieve Vragenlijst voor Psychosociale Problematiek bij Adolescenten.

^ll^CSGA: Cancer-Specific Geriatric Assessment.

^mm^CHQ-CF: Child Health Questionnaire-Child Form.

^nn^EQ-5D: European Quality of Life-5 Dimensions (General Health).

^oo^PHQ-9: Patient Health Questionnaire-9.

^pp^FFbH: Hannover Functional Ability Questionnaire.

^qq^BASDAI: Bath Ankylosing Spondylitis Disease Activity Index.

^rr^PGA: Patient Global Disease Activity.

^ss^ROAD: Recent-Onset Arthritis Disability Index.

^tt^TJC: tender joint count.

^uu^BASFI: Bath Ankylosing Spondylitis Functional Index.

^vv^NRS: numeric rating scale.

^ww^KDQOL-36: Kidney Disease Quality of Life Instrument.

^xx^ESAS: Edmonton Symptom Assessment System.

^yy^PLWH: people living with HIV/AIDS.

^zz^ODI: Oswestry Disability Index.

^1^NDI: Neck Disability Index.

^2^HOOS: Hip Disability and Osteoarthritis Outcomes Score.

^3^KOOS: Knee Injury and Osteoarthritis Outcomes Score.

^4^QuickDASH: abbreviated version of Disabilities of the Arm, Shoulder, and Hand.

^5^PSS: Perceived Stress Scale.

^6^FFI: Foot Function Index.

^7^None mentioned in particular.

### Methodological Quality

The risk of bias scores and the level of evidence, based on the classification of the Dutch Centraal BegeleidingsOrgaan-classificatiesysteem [52], are reported in Table 1. Scores ranged from 3/10 to 8/10 for the experimental studies and from 6/14 to 10/14 for the observational studies. Level A2 evidence was determined for 10 studies [32,35,36,38-40,42,44,46,50], level B for 12 studies [23,26,28,29,37,41,43,45,47,51], and level C for 10 studies [20-22,24,25,27,30,31,33,34].

### Benefits for Patients

#### Preference and Satisfaction

**Figure 2:**
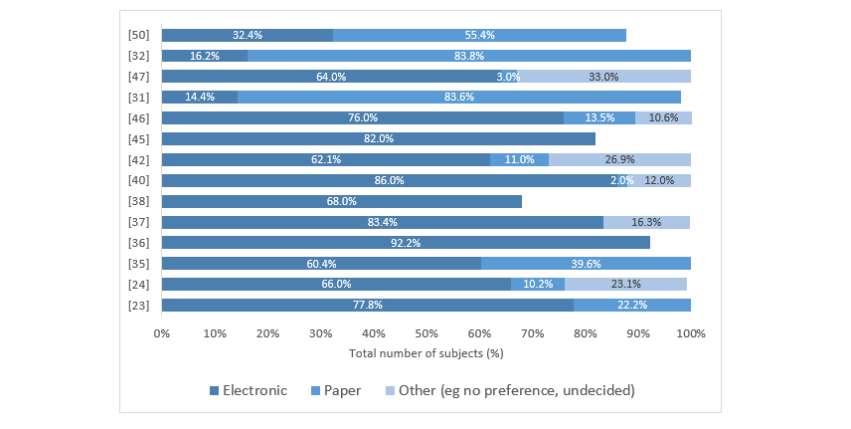
Preferred mode of form administration.

The preferred modality (electronic vs paper) was reported in 14 studies [23,25,31,32,35-38,40,42,45,50], and electronic administration was preferred in 11 studies [23,25,35-38,40,42,45-47]. One study reported a significantly greater preference for the tablet-delivered questionnaires (*z*=4.96, SE 3.428, *P*<.001) [49]. Another study asked patients to rate which mode of administration was the most pleasant and least difficult to use with a Likert scale [44]. Overall, of the 16 studies that reported user preference [23,24,31,32,35-38,40,42,44-47,49,50], a preference for ePROM was reported in 13 studies [23,24,35-38,40,42,44,49]. An overview of the reported percentages can be found in Figure 2.

Additionally, 4 [23,32,42,49] of the 16 studies reported sociodemographic variables that significantly influenced the preference for electronic administration (Table 3)*.*

**Table 3:** Sociodemographic variables influencing the preference for electronic patient-reported outcome measures.

Study	Population	Significantly preferred electronic patient-reported outcome measures
Engan et al 2016 [23]	Patients in cardiac, lung, occupational, and cancer rehabilitation programs	Younger age (*P*=.008), married/cohabitating (*P*=.004), internet availability (*P*<.001)
Richter et al 2008 [42]	Patients with rheumatoid arthritis, lupus, or spondyloarthritis	Younger age, better computer knowledge (*P*<.01)
Keurentjes et al 2013 [32]	Patients post-THR^a^ or TKR^b^	Younger age (*P*<.001), men (*P*<.001), higher education level (*P*<.001), higher BMI (*P*=.004)
Fanning et al 2014 [49]	Healthy aging adults (n=47)	Daily computer use (*r*_s_=.42, *P*<.05), perceived ease of use (*r*_s_=.665, *P*<.001), reported anxiety while completing digital questionnaires (*r*_s_=.552, *P*<.001)

^a^THR: total hip replacement.

^b^TKR: total knee replacement.

The satisfaction with and attitude towards ePROMs were reported in 7 studies. Most patients who were exposed to an ePROM found it easy to learn, easy to use, would recommend it to other patients, and would like to continue using it [20,21,33,34,43,47]. In a feasibility and acceptability study of a smartphone app for seizure self-management, patients with epilepsy thought ePROMs would reduce medical visits and health-related costs. Positive satisfaction levels with ePROMs were found for people who were younger (*P*=.002), lived in a city (*P*<.001), had higher education levels (*P*=.001), had stable employment (*P*<.001), had more frequent seizures (*P*=.01), had poor medication adherence, and owned a smartphone (*P*=.001) [21]. In breast cancer patients, willingness to use ePROM was higher in the group with previous experience with ePROM than in the group with previous experience with only paper PROM (92.3% and 59%, respectively, *P*=.001) [26]. Finally, reviewing the results with a health care professional was associated with 6.6-fold increased odds (*P*<.001) of perceiving systematic ePROMs as a benefit [33].

#### Completion Time

Time to complete electronic and paper-based questionnaires was reported in 9 studies [35-37,40,43,45-48], and 3 of these studies reported no significant differences in completion time [35,47]. In one study, however, subjects reported that the completion time for the electronic variant was more acceptable (*P*=.02) and was perceived as less of a barrier (*P*=.003) compared to the paper version [36]. Significantly lower times for the electronic variant were reported in 3 other studies [37,40,43]. Only 2 of the 9 studies reported significantly lower completion times for the paper version [45,46], owing to the longer log-on procedure required for the ePROM [45]. One study was indecisive. A detailed overview of the completion times can be found in Table 4. Overall, the completion times for ePROMs were at least equal to or faster than those for paper forms.

**Table 4:** Completion times for electronic questionnaires, compared with the paper-based counterpart.

Study and instrument		Time for electronic completion, mean	Time for paper completion, mean	*P* value	Remarks
**Shah et al 2016 [35]**					N/A
	EQ-5D^a^	88 s	81 s	.105	
	ODI^b^	145 s	143 s	.869	
	NDI^c^	124 s	117 s	.716	
	HOOS^d^	247 s	238 s	.829	
	KOOS^e^	255 s	259 s	.916	
	QuickDASH^f^	111 s	117 s	.723	
**Touvier et al 2010 [36]**					
	NutriNet-Sante anthropometric questionnaire	—^v^	—	.07	Time for electronic considered more acceptable (*P*=.02) and less a barrier (*P*=.003)
**Salaffi et al 2013 [37]**					
	BASDAI^g^, BASFI^h^, NRS^i^	5.1 min	7.9 min	.04	Computer skills, age, and education had no impact (*P*>.05)
**Salaffi et al 2009 [40]**					
	VAS^j^ GH^k^, VAS Pain, VAS PGA^l^, ROAD^m^, TJC^n^	7.3 min	7.9 min	.006	Older age was associated with slower times for both electronic (*P*=.02) and paper (*P*=.005)
**Hollen et al 2013 [43]**					
	LCSS^o^	2.2 min	3-5 min	N/A	N/A
**Bliven et al 2001[45]**					Not significant without the time for the log-on procedure
	SAQ^p^	5.53 min	4.78 min	<.05	
	SF-36^q^	6.76 min	5.44 min	<.05	
**Ali et al 2017 [46]**					
	DLQI^r^	78 s	73 s	.008	Older age was associated with longer time (*r*^2^=.257, *P*=.012)
**Greenwood et al 2006 [47]**					
	RAQol^s^	5 min	6 min	.194	N/A
**Kesterke et al 2015 [48]**					When data entry is added, WOMAC electronic signature was faster (*P*<.001) and no difference for FJS (*P*=.169)
	WOMAC^t^	117 s	170 s	<.001	
	FJS^u^	37 s	22 s	<.001	

^a^EQ-5D: EQ-5D: European Quality of Life-5 Dimensions (General Health).

^b^ODI: Oswestry Disability Index.

^c^NDI: Neck Disability Index.

^d^HOOS: Hip Disability and Osteoarthritis Outcomes Score.

^e^KOOS: Knee Injury and Osteoarthritis Outcomes Score.

^f^QuickDASH: abbreviated version of Disabilities of the Arm, Shoulder, and Hand.

^g^BASDAI: Bath Ankylosing Spondylitis Disease Activity Index.

^h^BASFI: Bath Ankylosing Spondylitis Functional Index.

^i^NRS: numeric rating scale.

^j^VAS: visual analogue scale.

^k^GH: global health.

^l^PGA: Patient Global Disease Activity.

^m^ROAD: Recent-Onset Arthritis Disability Index.

^n^TJC: tender joint count.

^o^LCSS: Lung Cancer Symptom Scale.

^p^SAQ: Seattle Angina Questionnaire.

^q^SF-36: Short Form-36.

^r^DLQI: Dermatology Life Quality Index.

^s^RAQol: Rheumatoid Arthritis Quality of Life Questionnaire.

^t^WOMAC: Western Ontario and McMaster Universities.

^u^FJS: Forgotten Joint Score.

^v^No statistically significant difference between ePROMs and paper PROMs in unanswered questions or complete questionnaires.

### Benefits for Health Care Workers or Centers

#### Cost

Engan et al [23] calculated and compared the human resource (HR) costs, specifically the time spent by an employee preparing, receiving, and handling data, of web-based and paper-based questionnaires. The mean HR cost for the web version was 9.5 minutes, whereas the mean HR cost for the paper version was 24 minutes.

Based on a cohort of 500,000 subjects [36], the financial costs of a paper-based questionnaire were calculated, including printing, mailing, returns, and double data entry. In total, it cost €4,965,833 (€9.94/subject) to use a paper-based version. In comparison, the development of a web-based tool by professionals was estimated to cost only €150,000 (€0.3/subject) or just 3% of the amount of the paper version.

Overall, these results indicate that digital data collection is less expensive, especially with large sample sizes, and it reduces HR-related costs.

#### Data Quality and Completion

Of the 10 studies [23,31,35,36,38,39,41,42,45,50] that reported on missing and incomplete data, 7 studies [23,31,36,38,39,41,50] indicated that electronic methods are associated with less missing data and more complete data. Integrated controls embedded in their ePROM administration was reported by 3 articles [23,35,36]. When a question wasn’t answered, an alert message provided the option to revise the answer prior to submission. As such, data entry mistakes in the form of missing, inconsistent, or abnormal values could theoretically be reduced to zero [23,36]. Regarding unanswered questions or incomplete questionnaires, 2 studies reported no statistically significant differences between ePROMs and paper PROMs [35,45]. One study [42] found significantly more missing items in the electronic version. And, one study reported that the answers were more detailed in 4 of 5 open questions on their electronic questionnaire (*P*<.05) [31]. Details of these results can be found in Table 5. Based on these results, we conclude that data quality is higher with ePROMs.

**Table 5:** Data quality of electronic questionnaires compared to their pencil-and-paper counterpart.

Study and missing data, unanswered questions, or incomplete forms								
Instrument and outcome unit			Electronic	Paper	*P* value		Remarks	
**Engan et al 2016 [23]**								
	**PAM-13^a^, MacNew^b^, FQ^c^, EORTC^d^, QLQ-C30^e^, HADS^f^**							
		Mean number of missing answers per patient	0.55	2.15	<.001		No inadequate responses in the web version due to integrated controls	
**Shah et al 2016 [35]**							No difference in unanswered questions	
	**EQ-5D^g^**							
		Mean number of unanswered questions	1.08	1.30	.083			
	**ODI^h^**							
		Mean number of unanswered questions	1.14	1.23	.619			
	**NDI^i^**							
		Mean number of unanswered questions	1	1.75	.541			
	**HOOS^j^**							
		Mean number of unanswered questions	6.7	5.5	.788			
	**KOOS^k^**							
		Mean number of unanswered questions	1.5	3.8	.220			
	**QuickDASH^l^**							
		Mean number of unanswered questions	1	1	1			
**Touvier et al 2010 [36]**						N/A^m^		Non-existent in web-based version due to integrated controls
	**NutriNet Sante questionnaire**							
		Data entry mistakes	0	82				
		Missing values	0	60				
		Inconsistent values	0	57				
		Abnormal values	0	3				
**Smith et al 2016 [38]**								
	**PSS^n^**							
		Incomplete forms	3	29	<.001		14 times more likely to be incomplete	
	**FFI^o^**							
		Incomplete forms	0	20	<.001		260 times more likely to be incomplete	
	**ODI**							
		Incomplete forms	1	10	<.001		11 times more likely to be incomplete	
**Raat et al 2007 [39]**								
	**CHQ-CF^p^**							
		Mean % missing answers per item	0.04%	0.54%	<.01		N/A	
**Dy et al 2012 [41]**								
	**DASH^q^**							
		Mean number of missing questions	0.1	2.6	<.001		N/A	
**Richter et al 2008 [42]**								
	**FFbH^r^, BASDAI^s^, SF-36^t^**							
		Number of missing items	NR^u^	NR	<.05		N/A	
**Bliven et al 2001 [45]**								
	**SAQ^v^**							
		Incomplete forms	5	5	N/A		N/A	
	**SF-36**							
		Incomplete forms	4	4	N/A		N/A	
**De Rada et al 2014 [31]**								
	**SAQ**							
		% unanswered questions	4.9%	9.3%	<.01		N/A	
**Kongsved et al 2007 [50]**								
	**SF-36, MFI-20^w^, HADS**							
		% complete forms	97.8%	63.4%	<.001		N/A	

^a^PAM-13: Patient Activation Measure short form.

^b^MacNew: MacNew Heart Disease Health-related Quality of Life questionnaire.

^c^FQ: Fatigue Questionnaire.

^d^EORTC: European Organization for the Research and Treatment of Cancer.

^e^QLQ-C30: Quality of Life Questionnaire Core 30.

^f^HADS: Hospital Anxiety and Depression Scale.

^g^EQ-5D: European Quality of Life-5 Dimensions (General Health).

^h^ODI: Oswestry Disability Index.

^i^HOOS: Hip Disability and Osteoarthritis Outcomes Score.

^j^KOOS: Knee Injury and Osteoarthritis Outcomes Score.

^k^QuickDASH: abbreviated version of Disabilities of the Arm, Shoulder, and Hand.

^l^BASDAI: Bath Ankylosing Spondylitis Disease Activity Index.

^m^N/A: not applicable.

^n^PSS: Perceived Stress Scale.

^o^FFI: Foot Function Index.

^p^CHQ-CF: Child Health Questionnaire-Child Form.

^q^DASH: Disabilities of the Arm, Shoulder, and Hand.

^r^FFbH: Hannover Functional Ability Questionnaire.

^s^BASDAI: Bath Ankylosing Spondylitis Disease Activity Index.

^t^SF-36: Short Form-36.

^u^NR: not reported.

^v^SAQ: Seattle Angina Questionnaire.

^w^MFI-20: Multidimensional Fatigue Inventory.

#### Response Rate, Adherence, and Compliance

A retrospective cohort analyzed the annual data from PROM non-completers. PROM monitoring was completed via paper until 2010, and in 2011, ePROMs were implemented. The initial rate of PROM non-completers was 43%-58%. This decreased to less than 20% since the implementation of ePROMs in 2011 [28]. One randomized controlled trial reported response rates of 17.9% in the internet group and 73.2% in the paper group. After sending a reminder, response rates were 64.2% and 76.5%, respectively (risk difference 12.2%, *P*=.002) [50]. Another study found no differences in completion rates between ePROMs and paper PROMs (*P*=.208) [35].

There is conflicting evidence on the effect of electronic data collection on response rates and adherence. Adherence to ePROM declines over time [20,29]. The opportunity to send automated reminders (eg, email or notification) to subjects can improve response rates and compliance [20,50].

#### Other Benefits

The role of ePROMs in symptom management and decision making was acknowledged in multiple studies. Andikyan et al [20] and Schnall et al [22] reported that electronic symptom self-reporting was important in clinical decision making. Automated data collection and processing via ePROM can generate automated alerts to health care professionals when a patient reports disturbing or severe symptoms [20]. It allows early detection of complications, immediate action, and potentially reduction in symptom burden, complications, and readmissions to the hospital. Furthermore, it empowers patients and improves patient-clinician communication [22,24,42]. This is facilitated by the opportunity to plot results visually with a graph or visual aids and gives both the patient and clinicians better insight in the evolution of the patient’s health status [25,34,43].

ePROMs have the advantage of always being available [24,25]. There is no paper waste [34,41], and ePROMs are portable and can be used to measure across multiple devices [42,46,49]. These reported ‘other benefits’ originate from studies with the lowest methodological quality.

### Disadvantages

As of May 25, 2018, all European organizations are expected to be compliant with the General Data Protection Regulations. This is reassurance for patients that the law is on their side when it comes to the use of their personal health data. All included articles and studies were performed before the implementation of the General Data Protection Regulations. However, privacy concerns were reported in 2 studies [21,26]. Liu et al [21] reported that the majority of patients (71.7%) thought their privacy should be adequately protected. In another study, patients were asked whether there were any barriers related to privacy and technology that would negatively influence their willingness to use ePROMs, and 30% were concerned about privacy issues. The study showed that barriers can be overcome by exposing the patients to an ePROM, which significantly influenced the willingness to participate in electronic assessments [26].

Disadvantages due to technical issues were addressed in 5 articles. The difficulty of or problems with login procedures were addressed in 3 studies [24,45]. Furthermore, technical difficulties adversely impacted compliance; patients who experienced technical difficulties completed fewer daily symptom entries (41.0%) than those who did not (76.0%) [29]. In another study, the needs and possible technological support structures were investigated. The importance of different possible support services to help complete a web-based questionnaire was assessed. Onsite support services were rated as being moderately or highly important by 38%. Technical telephone support was rated as moderately important or very important by 52%. At least 61% would appreciate receiving direct feedback after using the ePROM app [26].

Electronic data collection may require a large initial financial investment (eg, to purchase tablets or computer infrastructure and software, equipment costs, hiring computer programmers, or accessing cellular internet) [30,36,45].

A major disadvantage of ePROM is the potential of a ‘digital divide’. People who are computer illiterate, are older, or have no access to infrastructure could be disadvantaged. One study reported that more than 50% of >70 year olds were not able to complete the electronic version without assistance due to computer illiteracy; less assistance was required for patients completing the paper version [27]. In a second study, patients who needed support were significantly older [42]. The digital divide was also illustrated in another study with cancer patients. Patients who refused ePROM or chose phone calls over (home-based) ePROMs were approximately 10 years older. Patients may differ in terms of available internet, user experience, and affinity for new media. Older or computer-illiterate patients need opportunities to familiarize themselves with the devices [24]. Older patients with poorer health-related quality of life and fewer pre-existing technical skills reported barriers for ePROMs more frequently [26]. Wintner et al [24] reported that patients found ePROMs too impersonal.

### Suggestions

**Figure 3:**
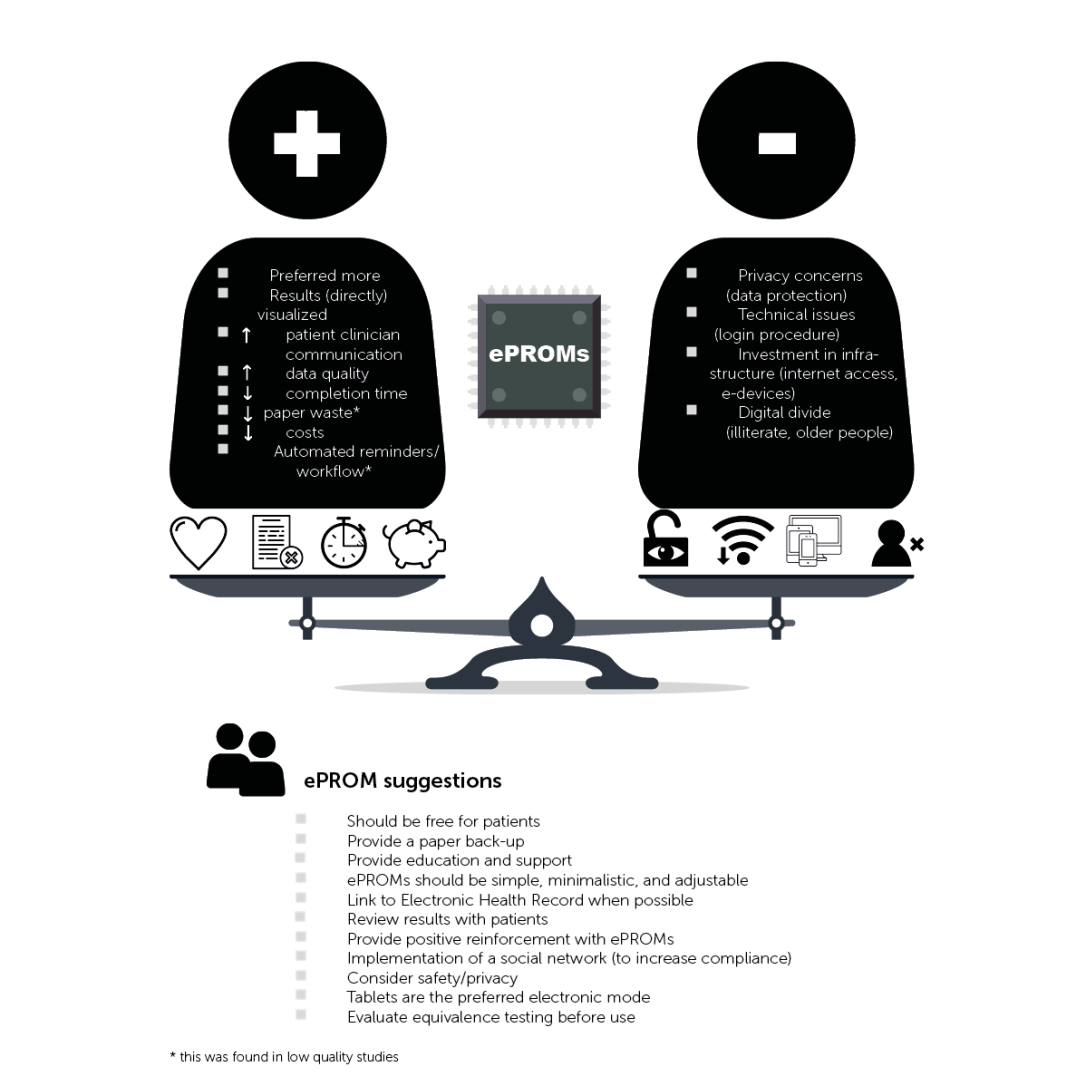
Comprehensive overview of the benefits of, disadvantages of, and suggestions for electronic patient-reported outcome measures (ePROMs).

Suggestions and tips for ePROM apps were extracted from 12 studies [20,21,24,27,29,30,32-35,38,42]. ePROMs should be free, simple, and minimalistic. They should have a good design, good user experience, adjustable font size, and adaptable user interface. When you start implementing ePROMs, provide educational sessions or support, think of the link with electronic health records, and review the results of the ePROMs with the patients because of the increased perception of benefit. ePROMs should provide positive reinforcement for the patients. Based on our results and discussion, we created a comprehensive overview of the benefits, disadvantages, and suggestions for ePROMs (see Figure 3).

## Discussion

### Principal Findings

The goal of this systematic review was to systematically and critically summarize the evidence on the use of ePROMs and find the potential benefits and disadvantages. We conclude that ePROM collection is feasible and accepted in healthy people and a wide range of patients with different conditions. Taking into account the results from the strongest methodological studies and the items that were reported in multiple studies, electronic data collection is preferred over paper-based collection, costs less, improves data quality, results in similar or faster completion times, and requires less administration time. Clinical decision making in combination with adequate symptom management can be facilitated. Expressed opinions reflected positive thoughts and attitudes towards ePROMs. Overall, participants found it easy to use, found it easy to learn, and would recommend it to others.

### Strengths and Limitations

Although our findings are generally favorable towards ePROMs, we cannot ignore the potential disadvantages. Aspects to consider are privacy protection, the one-time large financial investment, and exclusion of certain populations. Patients may be unwilling or unable to complete ePROMs due to higher age or computer illiteracy. Some patients have no internet access, do not have technological devices, or are not acquainted with technological devices. These reported disadvantages and barriers need to be considered when implementing a digital data collection tool in any population. Potential solutions may include an educational session on the use of the digital app and providing sufficient support [24,27,42]. It is also useful to at least provide back-up pen-and-paper data collection to avoid excluding segments of the population from receiving the best possible health care [20,32]. Several suggestions to keep in mind when creating an ePROM are also mentioned in this literature review, which could increase patient experience, usability, and acceptability.

Considering the influence of age, 2 studies suggest that it is an important factor that could potentially increase completion time [40,46]. In contrast, one study found no relationships between completion time and computer skills, age, or education [37]. Older people in particular have reservations concerning modern computer technology and need to be properly approached, especially since we found that younger people had a significantly greater preference for ePROMs [23,32,42]. In our systematic review, we found that various groups of patients with a chronic disease preferred ePROMs over paper versions. On the aspect of completion time, only the time for the patient to complete the questionnaire was measured in the included articles. However, one of the greatest reported advantages of electronic data collection is automated data processing [36,38,41-43,45,49], which subsequently reduces HR time [23], and data are less prone to administration errors. Clinical-based decision-making models using daily registration of PROMs can thus be created.

The strengths of this literature review are that 32 studies concerning the research question were retrieved. Not all studies were comparative trials but assessed patient satisfaction or attitude towards a single ePROM [21,24,25,32,33]. These studies, although not methodologically the strongest, provided capital insights for the research question.

In this systematic literature search, we only searched two databases. It is, therefore, possible that we missed some clinical studies. Moreover, the limited methodological quality of some of the included studies diminished the power of the recommendations.

The overall methodological quality of the included articles was moderate. Disadvantages were a lack of blinding of participants, heterogeneity of outcome measures, heterogeneity of patient populations, different ePROM questionnaires, and different ePROM modalities/formats. Generalizing or comparing results is therefore more difficult, and the results should be interpreted with caution.

The most frequently used screen-based device was tablets. This may be because tablet screens are larger than traditional handheld devices, are easy to use, and can be used for device-based systems. They can provide access to web-based portals or can be used with downloadable apps, which makes them the primary platform for site-based (ie, hospital, care centers) ePROM collection. On the contrary, desktops usually lack touch screen functionality and require the use of a keyboard and/or mouse to respond to questions [14]. Different electronic modes were used in the different articles. The advantages and disadvantages of the different electronic modes are difficult to conclude from this study. Contrasting evidence was found in previously published literature. Two reviews reported their concerns of equivalence between different electronic modes [8,10]; however, White et al [10] found small differences in the correlations, which were not significant regardless of the electronic mode used. In clinical trials, multiple modes of administration may be used, and new findings may be compared to findings that used a different electronic mode of data collection. Further research is warranted regarding the influence of the electronic mode on measurement equivalence. Our findings predominantly complement those from other published literature. Belisario et al [53] conducted a review to assess the impact of apps on the quality of survey questionnaire responses and reported contradictory results regarding completion times but acknowledged that apps might improve data completeness with more complete records than paper administration. Similar to our findings, they reported that there is not enough evidence that apps impact adherence to sampling protocols. Muehlhausen et al [9] conducted a meta-analysis on the equivalence of electronic and paper administration of PROMs and showed that ePROMs yielded comparable results to those of the paper-based variant. Their findings also confirmed the ISPOR taskforce’s conclusion that full psychometric testing of new ePROMs is not necessary for migrations with minor changes only [12]. For researchers and sponsors, this is a clinically and financially reassuring aspect that might facilitate the decision-making process to migrate from paper to digital data collection. The bring-your-own-device (BYOD) approach for ePROM data collection shows potential. BYOD allows participants to use their own computer device (eg, smartphone, tablet, laptop) to access and complete ePROMs [14]. However, there are still a number of issues (eg, software, security, ownership) that need to be resolved before BYOD becomes widely used.

### Future Work

The importance of PROMs is widely accepted. Collecting PROMs with paper-based questionnaires requires many subsequent time-consuming steps [45] that hamper wide implementation in daily care. Electronic collection of PROMs overcomes many of these steps. The potential to collect, score, analyze, visualize, and almost instantly review the results may facilitate workflow. Clinically, we believe ePROMs will improve the interchangeability of information between health care workers, patient-clinician communication, and patient care due to its always available nature. In addition, automated data processing in combination with targeted strategies (eg, automated alerts when patients report disturbing symptoms) has major clinical implications. Clinicians and researchers will also benefit from digital data collection since it reduces administration time. Furthermore, integration of ePROMs into electronic health records may be fundamental to advancing clinical care to improve patient engagement and health outcomes.

### Conclusion

Based on this study, we found multiple advantages for the use of ePROMS in several fields of care. ePROMs are preferred over paper-based forms, cost less, improve data quality, result in similar or faster completion times, reduce administration times, and facilitate clinical decision making in combination with adequate symptom management. Subjects expressed positive thoughts and attitudes towards electronic data collection. Potential disadvantages have been mapped but they are not of the magnitude to disregard ePROMs. Furthermore, suggestions have been provided to counteract the disadvantages. This review allows researchers and clinicians to consider both the advantages and disadvantages of selecting one mode over the other. While electronic modes offer advantages for all involved parties (eg, patients, hospitals, government), implementing (new) ePROMs requires careful considerations of the implications on the study population and may require additional steps (eg, provision of internet access, acquiring electronic devices) to include participants who would be excluded otherwise.

## Abbreviations

ACQ: Asthma Control Questionnaire
AQLQ: Asthma Quality of Life Questionnaire
BARSE: Barriers Self-Efficacy Scale
BASDAI: Bath Ankylosing Spondylitis Disease Activity Index
BASFI: Bath Ankylosing Spondylitis Functional Index
CBO: Centraal BegeleidingsOrgaan-classificatiesysteem
CCC: concordance correlation coefficient
CHQ-CF: Child Health Questionnaire-Child Form
CSGA: Cancer-Specific Geriatric Assessment
DASH: Disabilities of the Arm, Shoulder, and Hand
DLQI: Dermatology Life Quality Index
eHealth: electronic health
EORTC: European Organization for the Research and Treatment of Cancer
ePRO: electronic patient-reported outcome
ePROM: electronic patient-reported outcome measure
EQ-5D: European Quality of Life-5 Dimensions (General Health)
ESAS: Edmonton Symptom Assessment System
FFbH: Hannover Functional Ability Questionnaire
FFI: Foot Function Index
FJS: Forgotten Joint Score
FQ: Fatigue Questionnaire
GH: global health
HADS: Hospital Anxiety and Depression Scale
HOOS: Hip Disability and Osteoarthritis Outcomes Score
HR: human resource
ICC: intraclass correlation coefficient
ISPOR: International Society for Pharmacoeconomics and Outcomes Research
KDQOL-36: Kidney Disease Quality of Life Instrument
KIVPA: Korte Indicatieve Vragenlijst voor Psychosociale Problematiek bij Adolescenten
KOOS: Knee Injury and Osteoarthritis Outcomes Score
LCSS: Lung Cancer Symptom Scale
LMSQoL: Leeds Multiple Sclerosis Quality of Life
MacNew: MacNew Heart Disease Health-related Quality of Life questionnaire
MFI-20: Multidimensional Fatigue Inventory
MFIS-5: Modified Fatigue Impact Scale-5
MMAS-8: Morisky Medication Adherence Scale
MSIP: Multiple Sclerosis Impact Profile
MSQoL-54: Multiple Sclerosis Quality of Life-54
NDI: Neck Disability Index
NRS: numeric rating scale
ODI: Oswestry Disability Index
OR: odds ratio
PAM-13: Patient Activation Measure short form
PASE: Physical Activity Scale for the Elderly
PDA: personal digital assistant
PGA: Patient Global Disease Activity
PHQ-9: Patient Health Questionnaire-9
PLWH: people living with HIV/AIDS
PRISMA: Preferred Reporting Items for Systematic Reviews and Meta-Analyses
PRO: patient-reported outcome
PROM: patient-reported outcome measure
PSQI: Pittsburgh Sleep Quality Index
PSS: Perceived Stress Scale
QLQ-C30: Quality of Life Questionnaire Core 30
QuickDASH: abbreviated version of Disabilities of the Arm, Shoulder, and Hand
RAQol: Rheumatoid Arthritis Quality of Life Questionnaire
ROAD: Recent-Onset Arthritis Disability Index
RQLQ: Rhinoconjunctivitis Quality of Life Questionnaire
SAQ: Seattle Angina Questionnaire
SF-36: Short Form-36
THR: total hip replacement
TJC: tender joint count
TKR: total knee replacement
VAS: visual analogue scale
WOMAC: Western Ontario and McMaster Universities
